# Continuous erector spinae plane block for robotic-assisted thoracic surgery in lung cancer: a dual-center retrospective propensity-score weighted cohort study

**DOI:** 10.1016/j.bjane.2026.844770

**Published:** 2026-06-06

**Authors:** Matheus Arraes, Sara Amaral, Saullo Queiroz Silveira, Leopoldo Muniz da Silva, Rafael Sousa Fava Nersessian, Francisco Jose Lucena Bezerra, Manoel de Souza Neto, Caroline Machado Nunes, Helidea de Oliveira Lima, Fernando Nardy Bellicieri, Glenio B. Mizubuti, Rafael Lombardi

**Affiliations:** a São Luiz Hospital – ITAIM/Rede, Department of Anesthesiology, Brazil; b Duke University Medical Center, Department of Anesthesiology, Durham, NC, USA; c Vila Nova Star Hospital/Rede D’Or, Department of Anesthesiology, São Paulo, SP, Brazil; d D'Or Institute for Research and Education (IDOR), São Paulo, SP, Brazil; e Queen’s University, Department of Anesthesiology and Perioperative Medicine, Kingston, ON, Canada; f University of Nebraska Medical Center, Department of Anesthesiology, Omaha, NE, USA

**Keywords:** Analgesia, Erector spinae plane block, Lung neoplasms, Propensity score, Robotic surgical procedures, Thoracic surgical procedures

## Abstract

**Background:**

Postoperative analgesia remains challenging in thoracic surgery, particularly with the rise of Robotic-Assisted Thoracoscopic Surgery (RATS). The Erector Spinae Plane Block (ESPB) is a promising opioid-sparing technique, but its role in RATS is unclear.

**Methods:**

In this retrospective dual-center cohort study, we included 78 patients with lung cancer undergoing RATS, who received general anesthesia with or without ESPB. ESPB involved a single shot of 30 mL of 0.5% ropivacaine injection at T7, followed by a 24-hour infusion of 0.2% ropivacaine (7 mL.h⁻¹). The primary outcome was opioid rescue within 36 hours. Secondary outcomes included hospital length of stay and chest tube duration. Confounders were balanced with Inverse Probability Treatment Weighting (IPTW).

**Results:**

In unadjusted analyses, there was no significant difference in rescue opioid consumption between groups (ESPB 37.8% vs. GA 27.3%; p = 0.46). After propensity score weighting to improve covariate balance, ESPB use was associated with lower postoperative opioid requirements (OR = 0.57; 95% CI 0.32–0.79; p = 0.01). This finding may reflect improved adjustment for measured confounders, although residual confounding cannot be excluded. In contrast, chest tube presence within 24-hours was associated with higher opioid requirements (OR = 2.32; 95% CI 1.22–4.20; p = 0.04). ESPB patients also required fewer scheduled opioids during the first 24–36h postoperatively (p = 0.03).

**Conclusion:**

In this observational cohort, ESPB use was associated with lower postoperative opioid requirements in RATS. These findings suggest a potential role for ESPB within ERAS pathways, although randomized studies are needed to confirm efficacy and define its role in minimally invasive thoracic surgery.

## Introduction

Postoperative pain management remains a critical challenge in thoracic surgery, in which inadequate analgesia can lead to severe complications, including atelectasis, pneumonia, respiratory failure, and chronic pain.^1^ Over the past decade, minimally invasive techniques such as Video-Assisted Thoracic Surgery (VATS) and Robotic-Assisted Thoracoscopic Surgery (RATS) have largely replaced traditional thoracotomy, reducing surgical trauma and enhanced recovery.^2^, ^3^, ^4^, ^5^ However, optimal pain control strategies must evolve alongside these advancements.

While thoracic epidural analgesia has long been considered the gold standard for thoracic procedures, its use has gradually declined due to well-recognized adverse effects, including hypotension and urinary retention, particularly when continuous local anesthetic-opioid combinations are employed.^6^ In this context, opioid-sparing multimodal analgesia strategies and ultrasound-guided fascial plane blocks have gained increasing attention. Among these, the Erector Spinae Plane Block (ESPB) has emerged as a promising alternative.^4^, ^5^, ^6^, ^7^ First described in 2016,^4^ ESPB is characterized by technical simplicity, a favorable safety profile, and a lower risk of neuraxial-related complications compared with traditional epidural techniques.^6^

Despite its growing adoption, key uncertainties remain regarding ESPB’s mechanism of action. Studies report inconsistent spread to the paravertebral, epidural, and intercostal spaces, with significant variability in block distribution among patients.^7^, ^8^, ^9^, ^10^, ^11^, ^12^, ^13^, ^14^ This raises questions about its reliability and optimal application, particularly in RATS, where evidence remains scarce.

Evidence specifically addressing the use of ESPB in RATS for lung cancer remains very limited, particularly regarding continuous ESPB techniques using catheter-based local anesthetic infusion. Available data are largely restricted to small case series or extrapolated from non-robotic or single-injection studies, leaving an important gap in evidence for this increasingly common surgical setting.^15^, ^16^, ^17^

In this context, the primary aim of this study was to evaluate the effectiveness of continuous ESPB as part of a multimodal, opioid-sparing analgesic strategy in patients undergoing RATS lung resection using a dual-center retrospective cohort design with propensity score weighting. We hypothesized that adding continuous ESPB to general anesthesia would reduce the proportion of patients requiring postoperative rescue opioids within 36 hours, compared with general anesthesia alone.

## Methods

This investigation was approved by the Institutional Research Ethics Board (IREB) (protocol 4.518.838, CAAE 42052821.2.0000.0087), which waived informed consent. STROBE guidelines were followed, and a checklist is attached in the supplementary material. This study complied with Resolution 466/2012 of the Brazilian National Health Council. The authors received no financial support for the research, authorship, and/or publication of this article. No funding bodies had any role in the design of the study; in the collection, analysis, or interpretation of data; in the writing of the manuscript; or in the decision to publish the results.

In this retrospective cohort, we reviewed electronic medical records at two tertiary hospitals between March 2019 and March 2022. All consecutive patients who underwent RATS for lung cancer during the study period were screened for eligibility. A total of 86 patients were initially identified. Six patients were excluded due to incomplete medical records, primarily related to missing postoperative pain assessments and/or incomplete documentation of rescue opioid administration, which precluded reliable classification of the primary outcome. One patient who received thoracic epidural analgesia and one patient with unintended ESPB catheter dislodgement were also excluded. No intraoperative conversions to open thoracotomy occurred during the study period. Patients with incomplete medical records were excluded prior to analysis, ensuring complete data availability for all covariates and outcome variables. No partial missingness remained, and therefore complete case analysis was performed without imputation.

During the study period, robotic thoracic surgeries were managed by a dedicated anesthesia team that routinely covered all eligible cases. The assignment of anesthesiologists to operating rooms was determined by standard departmental scheduling, independent of patient characteristics and without prior knowledge of which specific surgical cases would be performed. As a result, ESPB was not selectively offered based on individual patient factors or perceived pain risk.

For patients included in this study, thoracic procedures were consistently performed by the same group of anesthesiologists, who were instructed to follow an institutional protocol consisting of either general anesthesia alone or general anesthesia combined with ESPB. Although the study design was neither blinded nor systematically randomized, anesthesiologists became aware of individual case details only after assignment. This approach aimed to reduce selective application of ESPB, although residual confounding by indication cannot be entirely excluded.

We included patients aged ≥ 18 years, undergoing RATS with GA, with and without ESPB. We excluded patients with chronic opioid consumption, chronic pain, substance abuse, emergency surgeries, palliative care, Patient-Controlled Analgesia (PCA) with the installation of an epidural pump, single-shot epidural anesthesia, catheter dislodgement in patients undergoing ESPB, patients who were not extubated within 12 hours after surgery, combined surgical procedures, reoperation, comatose patients, those with impaired ability to assess pain or request analgesia, and incomplete medical records.

The primary outcome was the incidence of postoperative opioid rescue within 36 hours after awakening from the GA. Rescue opioids were administered only if patients experienced moderate to severe pain, either at rest or with movement. Pain was assessed using the Numerical Rating Scale (NRS), where 0 indicates no pain and 10 indicates extreme pain. Scores ≥ 4 were considered moderate or severe pain. The secondary outcomes included hospital length of stay and chest tube duration (in days). Although the NRS was used clinically to guide rescue analgesia according to a standardized institutional protocol, pain scores were not consistently documented in a structured manner in some of the medical records, and in some cases only analgesic administration was recorded. Therefore, longitudinal analysis of NRS values was not feasible, and the occurrence of rescue opioid administration was used as a surrogate marker of clinically significant pain.

The studied variables included age, gender, Body Mass Index (BMI), comorbidities, anesthetic technique, type of surgery, duration of surgery, postoperative use of opioids and pregabalin, chest tube duration, and Intensive Care Unit (ICU) length of stay.

The general anesthetic management and block technique were based on our hospital’s standard of care. Anesthetic management followed institutional standards and was individualized when clinically indicated, according to the judgment of the attending anesthesiologist.

Anesthesia was induced with propofol 2 mg.kg^-1^, and selective tracheal intubation was facilitated with rocuronium 0.6–1.2 mg.kg^-1^. Fentanyl or methadone was used as the opioid for induction, with dexmedetomidine and magnesium sulfate administered as analgesic adjuncts. Anesthesia was maintained with continuous infusions of propofol and remifentanil; propofol was titrated to maintain Bispectral Index (BIS) values between 40 and 60, while remifentanil was administered to provide intraoperative analgesia according to clinical judgment. As part of a standardized multimodal analgesia protocol, parecoxib 40 mg, pantoprazole 40 mg, and dexamethasone 0.1 mg.kg^-1^ (maximum 10 mg) were administered before surgical incision. Twenty minutes before surgical completion, intravenous metamizole 2000 mg was given.

Intraoperative monitoring consisted of Non-Invasive Blood Pressure (NIBP), ECG, Pulse Oximetry (SpO_2_), capnography, temperature, and BIS. Controlled ventilation was performed in a closed circuit with a flow up to 2 L.min^-1^, tidal volume up to 7 mL.kg^-1^, with a Positive end Expiratory Pressure (PEEP) of 6‒8 cm H_2_O, and respiratory rate to maintain an end-tidal CO_2_ between 35‒40 mmHg. Neuromuscular blockade was maintained at a post-tetanic count < 5 (i.e., deep block). Reversal of neuromuscular blockade with sugammadex was performed in all patients. A convective air circulation heating system maintained normothermia. The same surgical team conducted all the included surgeries.

The ESPB and catheter placement were performed at the end of surgery, before emergence from general anesthesia and prior to tracheal extubation, under sterile conditions and ultrasound guidance. The block was intentionally performed at this time point to avoid interference with intraoperative management and patient positioning, as preoperative placement of a continuous ESPB catheter could interfere with the surgical field during robotic thoracic surgery and increase the risk of unintended catheter displacement. Given the use of a continuous catheter, the primary aim of the block was postoperative analgesia rather than preemptive intraoperative analgesia.

The ESPB was performed in a standardized manner using a high-frequency linear ultrasound transducer. A single-shot unilateral ESPB was administered by injecting 30 mL of 0.5% ropivacaine into the fascial plane between the deep surface of the erector spinae muscle and the transverse process of the seventh thoracic vertebra. A 20-gauge epidural catheter was then inserted through the Tuohy needle and advanced 5 cm into the fascial plane. Continuous infusion of ropivacaine 0.2% was administered via the ESPB catheter at a basal rate of 7 mL.h^-1^, with the option of up to two additional bolus doses of 5 mL.h^-1^, for the first 24 hours. After this period, the catheter was removed, and analgesia continued with standard or rescue analgesics at the discretion of the surgical team.

The surgical team determined postoperative analgesic management. The regimen included oral oxycodone 10 mg every 12 hours, IV tenoxicam 40 mg every 24 hours, and IV metamizole 50 mg.kg^-1^ every 6 hours.

Pain was assessed at 15-minute intervals during the first postoperative hour and then every 6 hours after discharge from the Post-Anesthesia Care Unit (PACU). Patients were instructed to promptly inform the nursing staff if they experienced moderate to severe pain. Intravenous morphine was administered as rescue analgesia for both groups when the pain score was ≥ 4, with dosing set at 1 mg every 10 minutes for moderate pain (scores 4‒7) and 2 mg for severe pain (> 7), aiming to maintain a score below 4. Rescue opioid administration followed a standardized institutional protocol applied uniformly in both the Post-Anesthesia Care Unit (PACU) and the surgical ward. Nursing staff administered intravenous morphine based exclusively on patient-reported pain intensity using the NRS. Due to the presence of an indwelling ESPB catheter, blinding of nursing staff to group allocation was not feasible. This lack of blinding may have introduced performance or detection bias; however, the use of objective pain thresholds (NRS ≥ 4) and a standardized rescue opioid protocol aimed to minimize subjective influence on analgesic administration.

### Statistical analysis

The sample size was based on the available data, i.e., all patients who underwent RATS between March 2019 and March 2022. No statistical power calculation was performed before the study.

Data distribution normality was evaluated through a normal Quantile–Quantile (Q–Q) plot. For categorical variables, the Pearson Chi-Squared test was applied, with partitioned Chi-square analyses performed when p < 0.05. Comparisons between two independent groups were conducted using the Mann-Whitney *U*-test when the dependent variable was ordinal or continuous but did not meet normality assumptions. Propensity Scores (PS) were used to calculate weights: 1/PS for patients in the treatment group (ESPB) and 1/(1–PS) for those in the control group (GA), following the method of Inverse Probability of Treatment Weighting (IPTW).

To mitigate confounding inherent to the retrospective design, a propensity score–weighted analysis using Inverse Probability of Treatment Weighting (IPTW) was performed to estimate Odds Ratios (OR) with corresponding 95% Confidence Intervals (95% CI). The propensity score was derived from covariates selected *a priori* based on biological plausibility and prior evidence of association with postoperative pain, opioid consumption, and treatment allocation. These included demographic variables (age, sex, and body mass index), baseline clinical status (ASA physical status, diabetes mellitus, arterial hypertension, and chronic obstructive pulmonary disease), surgical characteristics (type and duration of surgery), and perioperative analgesic exposure (intraoperative doses of fentanyl, methadone, dexmedetomidine, and magnesium sulfate). Variables with a direct impact on postoperative pain and analgesic demand, including chest tube presence/duration, length of ICU stay, and scheduled opioid prescriptions during the first 36 postoperative hours, were not incorporated. Covariate balance after weighting was assessed using absolute standardized differences, with values < 0.1 indicating adequate balance.

The propensity score model included only pre-treatment variables selected a priori based on clinical relevance and prior literature, namely demographic characteristics, comorbidities, surgical factors, and intraoperative analgesic exposure. Variables that occurred after treatment allocation or were potentially influenced by the intervention (e.g., presence of a chest tube within the first 24 hours, ICU stay, and postoperative analgesic prescriptions) were intentionally excluded from the propensity score model to avoid adjusting for mediators.

The final weighted outcome model (Table 1) was constructed to estimate the adjusted association between ESPB use and postoperative opioid rescue, and additionally included clinically relevant postoperative variables (e.g., chest tube presence within the first 24 hours) to account for factors directly associated with postoperative pain and opioid requirements. In particular, the presence of chest tube within the first 24 hours was included as it is a well-recognized contributor to postoperative pain and opioid consumption, although it was not included in the propensity score model as it may lie on the causal pathway between treatment and outcome.

**Table 1:** Summary of propensity weighted analysis.

	Propensity weighted analysis		
	Estimate (OR)	95% CI	p-value
**Groups**			0.01
GA	1.00		
ESPB	0.57	0.32 – 0.79	
**Dose of Fentanyl (mcg)**	0.96	0.91 – 1.08	0.06
**Dose of Methadone (mg)**	1.12	0.87 – 1.15	0.09
**Chest tube in the First 24h**	2.32	1.22 – 4.2	0.03

Estimates correspond to Odds Ratio (OR); CI, Confidence Interval; Weighted analysis evaluating the association between ESPB use and postoperative rescue opioid requirements.

† Postoperative rescue opioid requirement was defined as the occurrence of moderate to severe pain with the need for opioid analgesics (morphine) in the first 36 hours. General Anesthesia without Erector Spinae Plane Block (GA); General Anesthesia with Erector Spinae Plane Block (ESPB). The propensity score was estimated using a logistic regression model based on pre-treatment variables only (demographics, comorbidities, surgical characteristics, and intraoperative analgesic exposure). The weighted outcome model additionally included selected postoperative variables (e.g., chest tube presence within the first 24h) to account for clinically relevant factors associated with postoperative pain.

Absolute Standardized Differences (SD) were computed to evaluate residual imbalances in measured covariates after cohort weighting. A threshold of SD < 0.1 was interpreted as adequate covariate balance between groups. All p-values were two-sided, and p < 0.05 was considered statistically significant. Overlap assumptions were evaluated by visually inspecting the distribution of estimated propensity scores across treatment groups, confirming the presence of a common region of support. The distribution of inverse probability treatment weights was examined to identify potential instability or excessively large weights. Because no extreme weights were observed, weight truncation was not required. All statistical analyses were performed using R Statistical Software (v4.0.1, The R Foundation, Austria).

## Results

**Figure 1:**
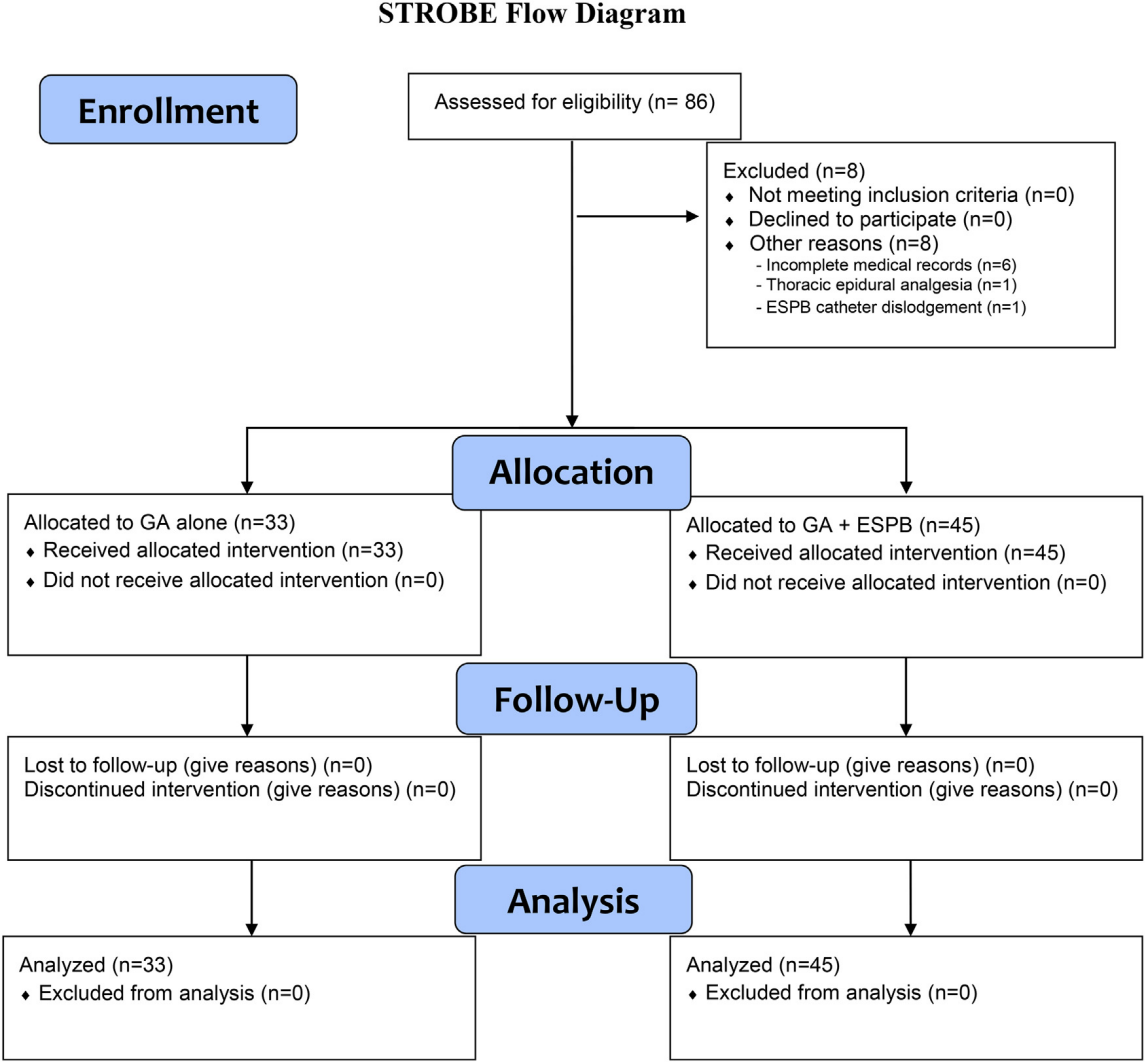
STROBE flow diagram illustrating patient screening, exclusions, and group allocation in this retrospective dual-center cohort study.

A total of 86 patients underwent RATS lung resection during the study period. After excluding 8 cases (6 for incomplete records, 1 epidural anesthesia case, and 1 ESPB catheter dislodgement), 78 patients were analyzed (45 in the ESPB group and 33 in the GA alone group). The cohort had a mean age of 62 ± 12 years and was 51.3% female. Demographic and clinical characteristics are detailed in Table 2. Surgical procedures included lobectomies (57.7%) and segmentectomies (43.3%) (Fig. 1).

**Table 2:** Patient characteristics, surgical procedures and outcomes.

**Age** (years)	62.62 ± 12.69
**BMI** (kg.m^-2^)	28.01 ± 5.14
**ASA-PS Classification** % (n)	
I	3.8 (3)
II	78.2 (61)
III	17.9 (14)
**Groups** % (n)	
General Anesthesia without Erector Spinae Plane Block (GA)	42.3 (33)
General Anesthesia with Erector Spinae Plane Block (ESPB)	57.7 (45)
**Diabetes** % (n)	
No	79.5 (62)
Yes	20.5 (16)
**Chronic Obstructive Pulmonary Disease (COPD)** % (n)	
No	85.9 (67)
Yes	14.1 (11)
**Arterial Hypertension** % (n)	
No	44.9 (35)
Yes	55.1 (43)
**Type of surgery** % (n)	
Thoracic Segmentectomy	42.3 (33)
Thoracic Lobectomy	57.7 (45)
**Surgical duration** (hours)	2.71 ± 1.02
**Intensive Care Unit (ICU) in the Postoperative Period** % (n)	
No	83.1 (64)
Yes	16.9 (13)
**Duration of chest tube** (Days)	1.88 ± 1.72
**Chest tube in the** f**irst 24h** % (n)	
No	44.9 (35)
Yes	55.1 (43)
**Chest tube in the first 48h** % (n)	
No	70.5 (55)
Yes	29.5 (23)
**Rescue Opioids in the first 36h** % (n)	
No	66.7 (52)
Yes	33.3 (26)
**Length of Hospital Stay** (Days)	3.39 ± 2.69

**Figure 2:**
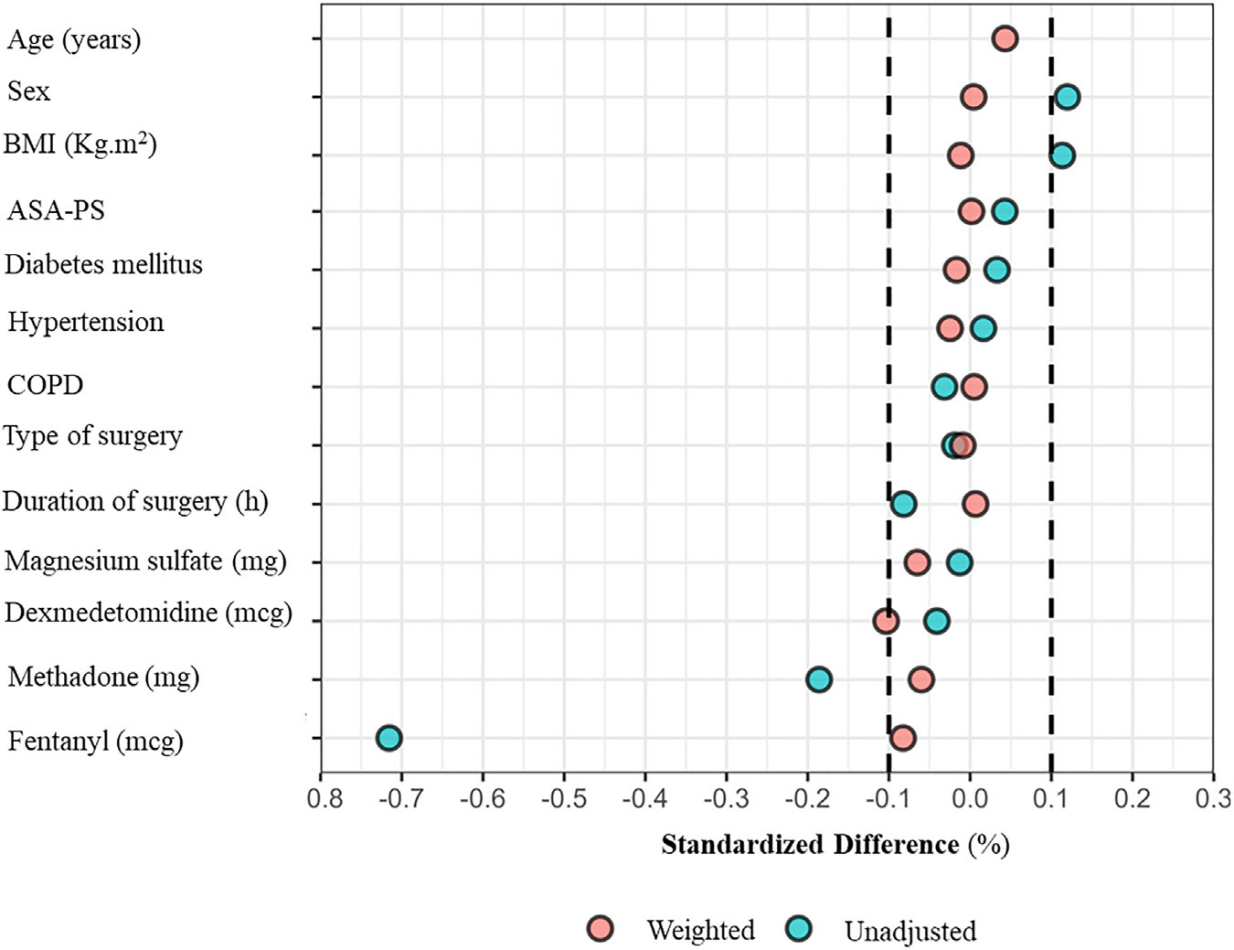
Standardized differences, prior to and following inverse probability-weighting. Abbreviations: Body Mass Index (BMI) (kg.m^-2^); Chronic Obstructive Pulmonary Disease (COPD); The American Society of Anesthesiologists Physical Status (ASA-PS classification).

The primary outcome occurred in 33.3% of patients (26/78) requiring rescue opioids within 36 hours. Unadjusted analysis showed no significant difference between groups (ESPB 37.8% vs. GA 27.3%, p = 0.46) (Table 3). Univariate regression analysis showed no statistically significant associations between postoperative rescue opioid requirements and the evaluated clinical variables; chest tube presence within the first 24 hours did not reach statistical significance (p = 0.08) (Table 4). Propensity-weighted analysis identified significant associations: ESPB use was associated with a lower likelihood of postoperative opioid rescue (OR = 0.57; 95% CI 0.32–0.79, p = 0.01), and chest tube presence within the first 24 hours was associated with increased odds of postoperative opioid rescue (OR = 2.32; 95% CI 1.22–4.20, p = 0.03) (Table 1). Based on marginal predicted probabilities derived from the IPTW-weighted model, ESPB was associated with a 9.7% absolute reduction (95% CI 3.0 to 16.0) in the risk of postoperative rescue opioid consumption within 36 hours. Covariate balancing after propensity score weighting yielded standardized differences that remained within accepted thresholds (Fig. 2).

**Table 3:** Patient characteristics, surgical procedures and outcomes of 78 patients undergoing robotic thoracic surgery according to whether they had received Erector Spinae Plane Block with general anesthesia (ESPB) or not (GA).

	Groups		
Variables	GA (33)	ESPB (45)	p-value
**Age (years)**^a^	65.0 ± 10.8	60.8 ± 13.7	0.15
**Gender %**(n)			0.88
Female	51.5 (17)	51.1 (23)	
Male	48.5 (16)	48.9 (22)	
**BMI (kg.m^-2^)**^a^	27.1 ± 5.3	28.6 ± 4.9	0.22
**ASA-PS classification**^b^			
**I**	0.0 (0)	6.7 (3)	0.31
**II**	81.8 (27)	75.6 (34)	
**III**	18.2 (6)	17.8 (8)	
**Hypertension**^b^	57.6 (19)	53.3 (24)	0.32
**Diabetes**^b^	27.3 (9)	15.6 (7)	0.88
**COPD**^b^	15.2 (5)	13.3 (6)	0.99
**Postoperative prescription of pregabalin**^b^	87.9 (29)	80.0 (36)	0.53
**Type of surgery**^b^			0.98
Thoracic Segmentectomy	42.4 (14)	42.2 (19)	
Thoracic Lobectomy	57.6 (19)	57.8 (26)	
**Surgical duration (hours)**^a^	2.60 ± 1.3	2.8 ± 0.78	0.38
**ICU in the postoperative period**^b^	27.3 (9)	9.1 (4)	0.07
**Duration of chest tube (days)**^a^	1.9 ± 1.8	1.8 ± 1.6	0.65
**Chest tube in the first 24 hours**^b^	51.5 (17)	57.8 (26)	0.75
**Chest tube in the first 48 hours**^b^	36.4 (12)	24.4 (11)	0.37
**Scheduled opioid prescription (first 24h)^#,^**^b^	72.7 (24)	33.3 (15)	0.001
**Scheduled opioid prescription (first 36h)^#,^**^b^	75.8 (25)	40.0 (18)	0.004
**Postoperative opioids rescue**^b^			0.46
No	72.7 (24)	62.2 (28)	
Yes	27.3 (9)	37.8 (17)	
**Dose of fentanyl**^c^**(mcg)**^a^	258.3 ± 97	235 ± 41.1	0.50
**Dose of methadone**^c^**(mg)**^a^	12.4 ± 4.8	13.8 ± 4.1	0.26
**Dose of dexmedetomidine**^c^**(mcg)**^a^	95.6 ± 69.5	105.5 ± 56.8	0.53
**Dose of magnesium sulfate**^c^**(mg)**^a^	4000 ± 1137.5	3555.6 ± 1318.8	0.22
**Hospital length of stay (days)**^a^	3.5 ± 2.3	3.2 ± 2.9	0.70

The American Society of Anesthesiologists Physical Status (ASA-PS classification); Confidence Interval (CI); General Anesthesia without Erector Spinae Plane Block (GA); General Anesthesia with Erector Spinae Plane Block (ESPB).

† Postoperative rescue opioid requirement was defined as the occurrence of moderate to severe pain with the need for opioid analgesics (morphine).

a Values expressed as mean ± standard deviation; #2 Standardized prescription with pre-defined scheduled dosing of oxycodone.

b Values expressed as % (n); p-values are based on regression analysis.

c Total cumulative dose of anesthetics administered during the intraoperative period.

**Table 4:** Patient characteristics, surgical procedures and outcomes of 78 patients undergoing curative robotic thoracic surgery according to the postoperative rescue opioids requirements (univariate regression analysis).

	Postoperative Rescue Opioids requirements^a^				
Variables	Total (n = 78)	No (n = 52)	Yes (n = 26)	OR (95% CI)	p-value
**Age (years)**	62.6 ± 12.6	63.2 ± 12	61.3 ± 12.5	0.99 (0.95 – 1.3)	0.54
**Gender**					
Female				1.00	
Male	48.7 (38)	53.8 (28)	38.5 (10)	0.54 (0.2 – 1.38)	0.22
**BMI (kg.m^-2^)**	28 ± 5.1	28 ± 4.5	27.8 ± 6.2	0.99 (0.9 – 1.09)	0.86
**ASA-PS Classification**					
**I**	3.85 (3)	3.8 (2)	3.8 (1)	1.00	
**II**	78.2 (61)	76.9 (40)	80.8 (21)	1.05 (0.1 – 23.37)	0.97
**III**	17.9 (14)	19.2 (10)	15.4 (4)	0.8 (0.06 – 20.11)	0.87
**Hypertension**	55.1 (43)	55.8 (29)	53.8 (14)	0.93 (0.36 – 2.4)	0.87
**Diabetes**	20.5 (16)	23.1 (12)	15.4 (4)	0.61 (0.15 – 1.98)	0.43
**COPD**	14.1 (11)	13.5 (7)	15.4 (4)	1.17 (0.28 – 4.31)	0.82
**Postoperative pregabalin prescription**	16.7 (13)	17.3 (9)	15.4 (4)	0.87 (0.22 – 3)	0.83
**Groups**					0.13
GA	42.3 (33)	46.2 (24)	34.6 (9)	1.00	
ESPB	57.7 (45)	53.8 (28)	65.4 (17)	1.62 (0.62 – 4.42)	
**Type of surgery**					0.15
Thoracic Segmentectomy	42.3 (33)	48.1 (25)	30.8 (8)	1.00	
Thoracic Lobectomy	57.7 (45)	51.9 (27)	69.2 (18)	2.08 (0.79 – 5.87)	
**Surgical duration (hours)**	2.58 (2.19–3.17)	2.58 (1.9–3.19)	2.71 (2.42–3.15)	1.17 (0.73 – 1.9)	0.50
**ICU in the postoperative period**	16.8 (13)	19.2 (10)	11.5 (3)	0.53 (0.11 – 1.96)	0.38
**Duration of Chest Tube (**d**ays)**	1.8 ± 1.7	1.9 ± 1.8	1.8 ± 1.6	0.99 (0.74 – 1.3)	0.96
**Chest tube in the first 24h**	55.1 (43)	48.1 (25)	69.2 (18)	2.43 (0.92 – 6.85)	0.08
**Chest tube in the first 48h**	29.4 (23)	38.4 (10)	25 (13)	1.87 (0.68 – 5.17)	0.22
**Schedule opioid prescription (**f**irst 24h)**^b^	50 (39)	50 (26)	50 (13)	1 (0.39 – 2.58)	1.00
**Schedule opioid prescription (**f**irst 36h)**^b^	55.1 (43)	53.8 (28)	57.7 (15)	1.17 (0.45 – 3.07)	0.75
**Dose of fentanyl (mcg)**	328.7 ± 229.8	346 ± 260.5	275 ± 65.4	0.48 (0.17 – 1.27)	0.15
**Dose of methadone**^c^**(mg)**	13.4 ± 4.3	12.9 ± 4.6	14.6 ± 3.6	1.1 (0.97 – 1.26)	0.16
**Dose of dexmedetomidine**^c^**(mcg)**	101.5 ± 61.8	100.5 ± 58.7	103.8 ± 69.4	1 (0.99 – 1.01)	0.84
**Dose of Magnesium Sulfate**^c^**(mg)**	3703.7 ± 1268.2	3562.5 ± 1389.6	3909 ± 1064.9	1 (0.99 – 1.11)	0.32

Body Mass Index (BMI) (kg.m^-2^); The American Society of Anesthesiologists Physical Status (ASA-PS classification); Confidence Interval (CI); General Anesthesia without Erector Spinae Plane Block (GA); General Anesthesia with Erector Spinae Plane Block (ESPB).

a Postoperative rescue opioid requirement was defined as the occurrence of moderate to severe pain with the need for opioid analgesics (morphine) in the first 36h.

b Standardized prescription with pre-defined scheduled dosing of oxycodone.

c Total cumulative dose of anesthetics administered during the intraoperative period. ^1^Values expressed as mean ± standard deviation. ^2^Values expressed as %(n); p-values are based on regression analysis.

Notably, the ESPB group received significantly fewer scheduled opioids in the first 24 hours (33% vs. 72%, p < 0.05) and 36 hours (40% vs. 75%, p < 0.05) (Table 3). Moreover, 42.2% of ESPB patients required no opioids on postoperative day one versus 9% in the GA group (p = 0.003). Early moderate-severe pain (first hour) occurred in 11.9% of cases, with no group difference (GA 14.8% vs. ESPB 10%, p = 0.83).

No significant intergroup differences were observed in intraoperative medications (fentanyl, methadone, dexmedetomidine, magnesium sulfate; all p > 0.20), hospital stay duration (mean 3.5 ± 2.4 GA vs. 3.3 ± 2.9 ESPB, p = 0.70), and chest tube days (mean 1.98 ± 1.82 GA vs. 1.80 ± 1.67 ESPB, p = 0.65) (Table 3).

## Discussion

The present dual-center retrospective study evaluated the effectiveness of the ESPB as part of an opioid-sparing strategy in patients undergoing RATS. Our findings suggest that ESPB use was associated with lower postoperative opioid requirements in the first 24 and 36 hours.

Epidural analgesia, traditionally considered the gold standard for thoracic procedures, presents well-documented complications, including hypotension, urinary retention, and technical difficulties, particularly in minimally invasive surgeries where the risk-benefit balance has shifted.^6^ Paravertebral blocks, although rare, also carry risks of pleural puncture and pneumothorax, which are less acceptable in the current surgical context.^6^ In this scenario, ESPB emerges as a promising alternative, with a favorable safety profile, ease of execution, and minimal risk of serious complications, as evidenced in large case series and systematic reviews.^5^^,^^14^^,^^18^

However, despite its increasing popularity, ESPB’s exact mechanism of action remains a subject of debate. Several cadaver and Magnetic Resonance Imaging (MRI) studies have demonstrated inconsistent spread patterns of local anesthetics, with variable diffusion into the paravertebral, epidural, and intercostal spaces.^7^^,^^12^ The lack of reliable paravertebral or epidural spread raises questions about the primary mechanisms through which ESPB exerts its analgesic effects. While MRI studies (e.g., Schwartzmann et al.,^8^ Sørenstua et al.)^12^ have confirmed some degree of paravertebral dissemination, clinical sensory blockade is often unpredictable.^13^ This suggests that mechanisms such as dampening of dorsal rami nociceptive input and systemic absorption of local anesthetics may also play significant roles in ESPB’s analgesic effect.

Our study contributes to this discussion by demonstrating that even in RATS, where surgical incisions often extend beyond the expected dermatomal coverage of ESPB, the block was associated with opioid-sparing outcomes. These findings are consistent with a prior case series by Cavaleri et al.^15^ and with the randomized clinical trial by Finnerty et al.,^16^ both of which reported improved postoperative analgesia with ESPB in minimally invasive thoracic procedures. Notably, Finnerty et al.’s randomized comparison between ESPB and Serratus Anterior Plane (SAP) block demonstrated superior opioid-sparing effects with ESPB, supporting its preferential use in thoracic surgery settings where deeper analgesia is desired.

Propensity score weighting was employed to balance confounders (e.g., age, comorbidities, intraoperative medications), isolating the effect of ESPB on rescue opioid requirements. The adjusted analysis showed that ESPB use was associated with 43% lower odds of requiring rescue opioids (OR = 0.57; 95% CI 0.32–0.79, p = 0.01) compared to General Anesthesia (GA) alone (reference group: OR = 1.00). Notably, this opioid-sparing effect persisted despite a comprehensive multimodal regimen that included systemic agents like dexmedetomidine, magnesium sulfate, and methadone, suggesting that ESPB may provide additive benefits beyond systemic pharmacologic interventions.

The discrepancy between the unadjusted and IPTW adjusted analyses was expected and likely reflected confounding inherent to the retrospective design and the non-randomized allocation of ESPB. Patients were not randomized to receive ESPB, and although they were not selected according to baseline clinical characteristics, the choice of multimodal analgesic strategy, including the decision to perform ESPB, was made at the discretion of the attending anesthesiologist. This clinical decision-making process, influenced by anticipated surgical pain and intraoperative considerations, may have contributed to differences in perioperative management that obscured the true association in the crude analysis. Variability in intraoperative opioid titration, use of adjunct analgesics, and postoperative prescribing practices may therefore have biased the unadjusted estimates toward the null. IPTW successfully improved covariate balance across measured pre-treatment factors, enabling a more accurate estimation of the independent association between ESPB and postoperative opioid requirements. Although the unadjusted analysis did not demonstrate statistical significance, the adjusted results were consistent with clinical plausibility and aligned with previously published evidence.^15^, ^16^, ^17^^,^^19^

Methadone should be specifically addressed in the Discussion due to its pharmacological properties, particularly its long elimination half-life and potential prolonged analgesic effects. In this study, methadone was administered as a single intraoperative dose and was similarly distributed between groups, providing a comparable baseline analgesic effect rather than differential exposure. Moreover, methadone dose was not independently associated with postoperative rescue opioid consumption and was adjusted for in the propensity score model. Notably, its concomitant administration in both groups may have provided sustained background analgesia over the early postoperative period, potentially attenuating differences in pain intensity between groups and, consequently, reducing the observed need for rescue opioids.

After adjustment, ESPB use was independently associated with a significantly lower likelihood of postoperative rescue opioid requirements, consistent with a clinically opioid-sparing association. These findings are in line with Enhanced Recovery After Surgery (ERAS) protocols for thoracic surgery, which emphasize opioid-sparing strategies aimed at enhancing recovery and minimizing opioid-related adverse events, such as respiratory depression, nausea, and ileus.^19^

Notably, scheduled opioid prescriptions in the first 36 hours differed significantly between groups (p = 0.004), reinforcing the importance of standardized postoperative protocols. The difference in scheduled opioid prescriptions likely reflects provider-driven analgesic strategies determined after treatment allocation, with ESPB use intentionally associated with reduced fixed opioid dosing. Although patients managed without ESPB and without scheduled opioids could theoretically require more rescue analgesia due to less robust baseline pain control, scheduled opioid prescription represents a post-treatment variable and is better interpreted as a mediator rather than a traditional confounder. Importantly, scheduled opioid consumption was not independently associated with rescue opioid requirements in univariate analysis (Table 4), suggesting that postoperative prescribing practices alone are unlikely to explain the adjusted opioid-sparing effect of ESPB, while a residual influence cannot be entirely excluded.

The lack of early (first hour) differences in moderate-to-severe pain incidence may be attributed to the multimodal analgesia administered intraoperatively, consistent with previous findings.^17^ Conversely, the presence of chest tubes in the first 24 hours significantly increased the odds of requiring opioid rescue, underscoring their well-known contribution to postoperative discomfort.^20^ While ESPB provided a protective effect, its ability to fully counteract chest tube-induced pain may be limited, reinforcing the need for comprehensive multimodal approaches.

### Limitations

This study has several limitations. Its retrospective design inherently introduced the risk of selection bias, information bias, and heterogeneity in perioperative management. Although propensity score weighting was used to mitigate measured confounding, residual confounding from unmeasured variables could not be fully excluded. Patients were not randomized to receive ESPB; instead, anesthesiology coverage was determined by standard departmental scheduling, which may have introduced differences in perioperative management practices across providers. Furthermore, the relatively small sample size may have increased the risk of unstable propensity score weights and model sensitivity, potentially influencing effect estimates. Despite efforts to minimize selective application of ESPB, confounding by indication related to the choice of ESPB versus general anesthesia alone remained a potential limitation inherent to this observational study.

Sensory block mapping was not performed, precluding direct correlation between dermatomal coverage and analgesic efficacy. Intraoperative opioid selection was not fully standardized; although methadone was similarly distributed between groups and adjusted for in the propensity score model, its prolonged and variable pharmacokinetics may have contributed to background analgesia in both groups. Scheduled postoperative opioid prescriptions represented post treatment variables and may have acted as mediators rather than traditional confounders, although they were not independently associated with rescue opioid requirements in univariate analysis. In addition, pain scores were not consistently documented in the medical records, and in some cases only analgesic administration was available, precluding detailed analysis of pain trajectories over time.

All analyses were conducted using complete case data because patients with incomplete medical records were excluded prior to analysis; although the number of exclusions was small, this approach may still have introduced selection bias and should be acknowledged as a methodological limitation. Finally, although no formal sensitivity analyses were performed due to sample size constraints, inspection of the propensity score distributions and inverse probability treatment weights did not suggest instability or undue influence of extreme weights. Nevertheless, the absence of additional formal robustness assessments should be considered when interpreting the results.

### Generalizability

The generalizability of these findings should be interpreted with caution. This retrospective analysis included a limited sample of patients undergoing elective RATS for lung cancer at two high-volume tertiary centers, all managed by experienced surgical and anesthesiology teams following ERAS-aligned protocols. The ESPB technique was standardized in terms of puncture level, dosing, and continuous infusion, which strengthens internal validity but may limit applicability to lower-volume institutions or settings with less expertise in ultrasound-guided regional anesthesia. Furthermore, the exclusion of emergency cases and patients with chronic pain or prior opioid consumption restricts extrapolation to more complex populations or larger thoracic procedures. Prospective multicenter studies are warranted to validate these findings across diverse practice environments.

## Conclusion

In this retrospective cohort, ESPB was associated with reduced postoperative opioid requirements in patients undergoing robotic thoracic surgery. Although these findings support ESPB as a potentially valuable component of multimodal, opioid-sparing analgesic strategies aligned with ERAS principles, causality cannot be established due to the observational design. Residual confounding and variability in perioperative analgesic management may have influenced the observed associations. Therefore, the role of ESPB in robotic thoracic surgery should be further evaluated in prospective randomized trials with standardized analgesic protocols, detailed sensory mapping, and long-term follow-up to confirm efficacy and clarify underlying mechanisms.

## Data availability statement

The datasets generated and/or analyzed during the current study are available from the corresponding author upon reasonable request.

## Declaration of generative AI and AI-assisted technologies

The authors declare that no generative artificial intelligence or AI-assisted technologies were used in the preparation of this manuscript.

## Disclosures

All authors (1) report no relationships that could be construed as a conflict of interest, (2) take responsibility for all aspects of the reliability and freedom from bias of the data presented and their discussed interpretation, and (3) have approved the final article.

## Authors’ contributions

All authors have made substantial contributions to (1) The conception and design of the work, (2) The acquisition, analysis, and interpretation of data, and (3) The manuscript draft and review of important intellectual content. Matheus Arraes, Sara Amaral, Saullo Queiroz Silveira, Leopoldo Muniz da Silva and Rafael Sousa Fava Nersessian were responsible for study conception, design, data screening/acquisition, analysis, interpretation, manuscript draft, and figures/tables creation. Caroline Machado Nunes contributed to study with data collection. Francisco Jose Lucena Bezerra and Manoel de Souza Neto contributed with manuscript/figures/tables review and manuscript draft. Glenio B. Mizubuti and Rafael Lombardi contributed with data interpretation/review, and manuscript draft.

## Conflicts of interest

The authors declare no conflicts of interest.
